# Ultrasound exfoliation of inorganic analogues of graphene

**DOI:** 10.1186/1556-276X-9-167

**Published:** 2014-04-05

**Authors:** Václav Štengl, Jiří Henych, Michaela Slušná, Petra Ecorchard

**Affiliations:** 1 Materials Chemistry Department, Institute of Inorganic Chemistry AS CR, v.v.i., Řež 250 68, Czech Republic; 2 Faculty of Environment, J.E. Purkyně University in Ústí nad Labem, Ústí nad Labem 400 96, Czech Republic

**Keywords:** Ultrasound, Exfoliation, Graphene inorganic analogues

## Abstract

High-intensity ultrasound exfoliation of a bulk-layered material is an attractive route for large-scale preparation of monolayers. The monolayer slices could potentially be prepared with a high yield (up to 100%) in a few minutes. Exfoliation of natural minerals (such as tungstenite and molybdenite) or bulk synthetic materials (including hexagonal boron nitride (h-BN), hexagonal boron carbon nitride (h-BCN), and graphitic carbon nitride (g-C_3_N_4_)) in liquids leads to the breakdown of the 3D graphitic structure into a 2D structure; the efficiency of this process is highly dependent upon the physical effects of the ultrasound. Atomic force microscopy (AFM), transmission electron microscopy (TEM), and selected area electron diffraction (SAED) were employed to verify the quality of the exfoliation. Herein, this new method of exfoliation with ultrasound assistance for application to mono- and bilayered materials in hydrophobic and hydrophilic environments is presented.

## Background

**Figure 1:**
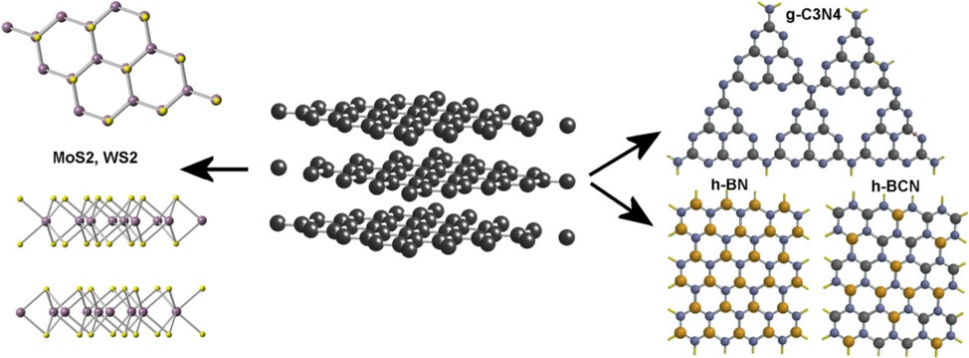
**The structures of inorganic analogues of graphene - MoS**_**2**_**, WS**_**2**_**, g-C**_**3**_**N**_**4**_**, h-BN, and h-BCN.**

Mechanical exfoliation, called the ‘scotch tape method’ [1], was the first method used for the preparation of single-layer graphene from natural graphite. Subsequently, through the utilization of this principle, other layered materials that are so-called inorganic analogues of graphene (IAG), such as MoS_2_[2,3] and WS_2_[4], hexagonal boron nitride (h-BN) [5], hexagonal boron carbon nitride (h-BCN), and graphitic carbon nitride (g-C_3_N_4_) (see Figure 1), were exfoliated. The current state of knowledge about the synthesis of IAGs is gathered below.

Some recent attempts to obtain ultrathin MoS_2_ include the preparation of monolayered MoS_2_ flakes that were mechanically exfoliated from a piece of commercially available crystalline MoS_2_ sample by the scotch tape method [6]. Joensen et al. [7] exfoliated MoS_2_ into monolayers by intercalation with lithium followed by a reaction with water. Chemically exfoliated MoS_2_ was also prepared via lithium intercalation using a solution of butyllithium in hexane. However, this method resulted in loss of semiconducting properties of the pristine MoS_2_, due to the structural changes that occurred during Li intercalation [8,9]. Yao et al. [10] reported on a method for the fabrication of monolayers and multilayers of BN, MoS_2_, and graphene utilizing a combination of low-energy ball milling and sonication. Ball milling generates shear and compression, which can cleave the layered materials into the 2D nanosheets.

Exfoliated WS_2_ was also prepared using ultrasonic treatments with *n*-butyllithium in hexane; this process was more difficult than the exfoliation of MoS_2_[8,9] due to the resistance of the WS_2_ to intercalation [11,12]. Single layers of the transition metal dichalcogenides WS_2_, MoS_2_, and MoSe_2_ were formed in aqueous suspensions by lithium intercalation and exfoliation of the crystalline powders [13]. Rao and Nag [14] described the preparation of WS_2_ and MoS_2_ sheets by lithium intercalation followed by exfoliation of the layers by ultrasonication and chemical synthesis, where molybdic acid and tungstic acid, respectively, were treated with an excess of thiourea. Another approach, a mixed-solvent strategy, exploited a low-intensity ultrasonic treatment (ultrasonic bath) for the exfoliation of MoS_2_, WS_2_, and BN in ethanol/water mixtures [15]. This method is suitable for the preparation of approximately 1% dispersion of exfoliated particles. Direct exfoliation of the bulk powder materials using supercritical CO_2_ assisted with ultrasound also led to the single and few layers of BN, MoS_2_, and WS_2_. The effects of supercritical CO_2_ coupled with ultrasound played a key role in the exfoliation process [16]. Warner et al. [17] presented a relatively simple method to prepare thin few-layer sheets of h-BN with micrometer-sized dimensions using chemical exfoliation in the solvent 1,2-dichloroethane. Lin et al. [18] demonstrated that water is effective in the exfoliation of layered h-BN structures with the assistance of an ultrasonic bath and leads to ‘clean’ aqueous dispersions of h-BN nanosheets without the use of surfactants.

The h-BCN compounds were successfully synthesized by using hydrogen-free 1,3,5-trichlorotriazine and boron tribromide as reactants and metal sodium as reductant through a chemical reduction synthesis method at 450°C [19]. A facile approach has been developed to prepare B and N co-doped graphene with tunable compositions simply through the thermal annealing of graphene oxide in the presence of boric acid and ammonia [20].

Hernandez et al. [21] gathered interesting findings that included the utilization of the method of liquid-phase exfoliation, where the surface energy of the solvent was advantageously used to exfoliate graphite. The surface energy of graphene, which is approximately 70 mJ/m^2^, is in the upper range of surface energies for most solvents. That would imply that this method cannot be used for the exfoliation of IAGs because the surface energies of these materials have been determined to be considerably higher than that of graphene. For example, Weiss and Phillips [22] referred that the surface energies of transition metal dichalcogenides, such as MoS_2_ and WS_2_, are greater than 200 mJ/m^2^. Therefore, there would not be any suitable solvents, and the method of liquid-phase exfoliation could not be used for the exfoliation of IAGs.

Ultrasonic power, transferred into the liquid by means of a sonotrode (ultrasonic probe, horn), is dependent on sonotrode shape, material, and the end load. An acoustic power of approximately 50 W/cm^2^ can be transferred into water at an ambient pressure. In a well-tuned ultrasonic system, it can be assumed that the power transfer into the liquid is more than 95% of the input power, and 3% to 4% of the losses are thermal losses of the electrical components of the generator. The maximum achieved power at ambient pressure is approximately 300 W. A further increase of the ultrasonic power can be achieved by placing the ultrasonic horn in the pressurized reactor. For 1-kW power supply, the optimal pressure is approximately 4 bar; for 2 kW, a suitable pressure is approximately 6 to 7 bar. Higher pressures load the ultrasound tool too much, and the ultrasonic generator begins its inevitable falling out of resonance and its power decreases. A liquid denser than water (ethylene glycol, glycerol, etc.) also leads to a higher output power, thanks to a higher cavitation threshold.

When the liquid is exposed to intense ultrasound, the waves propagate through the liquid causing an alternating of high-pressure and low-pressure cycles that is dependent on the frequency of the electric generator. During the low-pressure cycle, high-intensity small vacuum bubbles are created, as the liquid vapor pressure is achieved. When the bubbles reach a certain size, they collapse strongly during a high-pressure cycle. During this implosion, very high pressures, high temperatures, and speed liquid jets are locally generated. This phenomenon is called cavitation [23]. The resulting hydrodynamic forces are able to disintegrate agglomerates and to mill particles in solution.

The ultrasonic vibrations are transferred into an elastic environment by spreading the longitudinal or transverse waves. Transverse waves cannot propagate in a gas or a liquid because there is no mechanism for driving the motion perpendicular to the propagation of the wave; thus, they are transformed into standing (stationary) waves by the ultrasonic horn. Stationary waves are able to vibrate lamellar particles, using the vibration to overcome van der Waals forces. As a result, lamellar particles are gradually peeled off to reveal individual sheets. The particle milling effect is based on intense ultrasonic cavitation, while delamination is caused by stationary waves. Increasing the density of the solvent or/and increasing the pressure of the solvent will also increase the cavitation threshold [24,25]. Through the selection of suitable reaction conditions and factors (sonotrode shape, intensity of ultrasound, solvent density, pressure, etc.), it is then possible to favor the process of delamination over grinding and milling. Delamination of layered minerals [26] by ultrasound was successfully used for the preparation of exfoliated mica [27] and kaoline [28] under atmospheric pressure. Pressurized batch ultrasonic reactors were also used to exfoliate graphite to graphene [29], which then served as the precursor for the composite materials of graphene-anatase [30] and graphene oxide-anatase [31]. It can then be theorized that the exfoliation of IAGs using power ultrasound in an environment of strong polar aprotic solvents in a pressurized batch reactor could be achieved through this procedure.

In this paper, we demonstrate simple and low-cost methods for the preparation of single- and few-layered nanosheets of inorganic analogues of graphene, MoS_2_, WS_2_, h-BN, h-BCN, and g-C_3_N_4_, using stationary ultrasound waves in a pressurized ultrasonic reactor. According to the available literature, an alkaline manganate solution for the exfoliation of IAGs [32] has not been used.

### Experimental

All of the chemicals used - potassium permanganate (KMnO_4_), potassium hydroxide (KOH), hydrochloric acid (HCl), boric acid (H_3_BO_3_), urea (CO(NH_2_)_2_), and melamine (C_3_H_3_N_6_) - were supplied by Sigma-Aldrich Company, Ltd. (St. Louis, MO, USA). The natural minerals tungstenite (WS_2_) and molybdenite (MoS_2_) were obtained from US Research Nanomaterials, Inc. (Houston, TX, USA) and from Rokospol Ltd. (Uherský Brod, Czech Republic), respectively.

### Preparation of bulk h-BN and h-BCN

The bulk h-BN was prepared from boric acid and urea by the modified method reported by Nag et al. [33]. This chemical method allows for the control of the number of layers through the composition of the starting feedstock because the number of BN layers decreases with increasing urea content in the reaction mixture. The boric acid and urea, in a molar ratio of 1:3, were dissolved in 100 ml of water and heated at 70°C until the full evaporation of water occurred. The dried crystal powder was heated at 950°C for 5 h under a nitrogen atmosphere.

To synthesize the h-BCN bulk compound [34], boric acid was mixed with melamine in the ratio of 1:2 in an agate mortar. The mixture was then heated in a beaker at 200°C for 1 h and subsequently at 300°C for an additional 2 h. The obtained precursor was heated under a nitrogen atmosphere at 1,300°C for 5 h.

### Preparation of bulk g-C_3_N_4_

The g-C_3_N_4_ was prepared by direct heating of 5 g melamine powder and was put into an alumina crucible with a cover [35]. The sample was heated at 580°C for 2 h with a heat rate of 10°C/min. After heating, a yellow powder of bulk g-C_3_N_4_ was obtained.

### Exfoliated samples in a hydrophobic environment

Exfoliated MoS_2_, WS_2_, h-BN, h-BCN, and g-C_3_N_4_ were prepared in a large quantity from synthesized bulk samples by using a high-intensity cavitation field in a pressurized ultrasound reactor (UIP2000 hd, 20 kHz, 2,000 W, Hielscher Ultrasonics, GmbH, Teltow, Germany). A portion of 0.75 to 1 g of the bulk sample was suspended in 120 ml of appropriate aprotic solvent (*N*-methyl-2-pyrrolidone, *N*,*N*-dimethylformamide, or dimethyl sulfoxide) and exposed to an intense cavitation field in a pressurized batch ultrasonic reactor for 20 min. The pressure of 6 bar was set in the reactor by means of an air compressor [29]. The exfoliation led to the formation of stable suspensions in the hydrophobic (organophilic) solvents.

### Exfoliated samples in a hydrophilic environment

The exfoliated IAGs stabilized in an aqueous solution were prepared through high-intensity ultrasound in a solution of KMnO_4_ in an alkaline environment. Generally, 1 g of IAG was mixed with 120 ml of an aqueous solution of 1.5 g KMnO_4_ and 24 g KOH in an ultrasonic reactor. The reactor was sealed and pressurized to 6 bar, and the reaction mixture was sonicated for 10 min. After irradiation, a suspension of IAG and MnO_2_ in a dark green solution of K_2_MnO_4_ was obtained. The suspension was neutralized to a pH = 7 to 8 by adding HCl to convert all higher-valent (III, IV, VI) manganese species contents to soluble Mn^2+^. The whole obtained reaction product was purified by dialysis using a Spectra/Por 3 dialysis membrane (Spectrum Laboratories Inc., Rancho Dominguez, CA, USA).

### Chemically exfoliated bulk h-BN

The method of producing chemically exfoliated h-BN is based upon the preparation of graphene oxide [36]. In a typical experiment, 0.75 g of h-BN bulk powder was dispersed in 60 ml of 96% H_2_SO_4_. Subsequently, 3 g of KMnO_4_ was added, and the reaction mixture was stirred under heating at 40°C continuously for 6 h. The obtained pink suspension was subsequently poured onto ice and mixed with 200 ml of 30% H_2_O_2_. The pink squash quickly changed to a white suspension, which was washed by decantation and centrifugation until it reached a pH ∼ 7.0.

### Characterization methods

Diffraction patterns were collected using a PANalytical X'Pert PRO diffractometer (Almelo, The Netherlands) equipped with a conventional X-ray tube (CuKα 40 kV, 30 mA, line focus) in the transmission mode. An elliptic focusing mirror with a divergence slit of 0.5°, an anti-scatter slit of 0.5°, and a Soller slit of 0.02 rad were used in the primary beam. A fast linear position-sensitive detector PIXcel with an anti-scatter shield and a Soller slit of 0.02 rad were used in the diffracted beam. All patterns were collected with steps of 0.013° and 500 s/step. A qualitative analysis was performed with the DiffracPlus Eva software package (Bruker AXS, Berlin, Germany) using the JCPDS PDF-2 database [37]. A water suspension of the sample material was placed onto a sample holder for transmission experiments and then covered with a Mylar foil (6 μm thick, DuPont Tejjin Films, Chester, VA, USA). Then, the second Mylar foil covers the sample to avoid losses. Finally, the sample holder was completed with a sample holder ring, making it ready for X-ray diffraction (XRD) experiments in transmission mode. The crystallite size, interlayer spacing, and number of h-BN and h-BCN layers were calculated by using the classical Debye-Scherrer equations [38,39].

Atomic force microscopy (AFM) images were obtained using a Bruker Dimension FastScan microscope. The samples for AFM measurement were prepared through the spin coating method. The samples were prepared by pipetting the exfoliated h-BN and h-BCN water suspensions onto the synthetic mica as an atomically smooth support and then were spin-coated at 6,000 rpm for 1 min. A silicon tip on a nitride lever was used with ScanAsyst (Bruker) in the air contact mode for resonance frequencies ranging from 50 to 90 kHz.

The morphology of the sample powders was inspected by transmission electron microscopy (TEM), and the crystal structure was analyzed by electron diffraction (ED) using a 300-kV JEOL 3010 (Akishima-shi, Japan). As a specimen support for the TEM investigations, a microscopic copper grid covered by a thin transparent carbon film was used. Both samples were studied in a bright field and by electron diffraction with a selecting aperture (selected area electron diffraction (SAED)) mode at an acceleration voltage of 200 kV.

## Results and discussion

The method of exfoliation of IAGs in the alkaline environment is based on a process also related to the phenomena of cavitation. The reaction mixture KMnO_4_ and KOH reacts at elevated temperatures and forms dark green unstable K_2_MnO_4_, which undergoes spontaneous, slow decomposition to MnO_2_:

(1) $$4\;{\mathrm{KMnO}}_{4}+4\;\mathrm{KOH}\to 4\;K_{2}{\mathrm{MnO}}_{4}+2H_{2}O+O_{2}$$

(2) $$2\;K_{2}{\mathrm{MnO}}_{4}+2\;H_{2}O\to 2\;{\mathrm{MnO}}_{2}+4\;\mathrm{KOH}+O_{2}$$

The reaction suspension absorbs ultrasonic waves, causing a heating of the solution to a suitable reaction temperature above 60°C, which is necessary for the formation of alkali metal manganates and which also accelerates its decomposition according to Equations 1 and 2. Manganate solutions can intercalate IAGs, and oxygen species formed in these reactions could be helpful for exfoliation. The exfoliation processes based on longitudinal and stationary ultrasonic waves take place simultaneously. This method has proven to be very useful as a general method for exfoliation of available materials with a lamellar structure.

**Figure 2:**
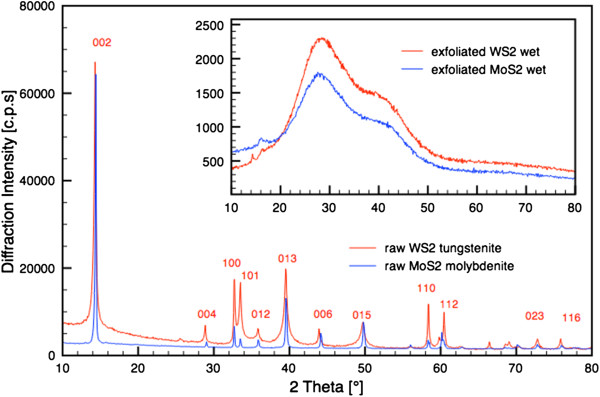
**XRD patterns of raw molybdenite MoS**_**2 **_**and tungstenite.** The inset shows the patterns of the ultrasound-exfoliated samples.

**Figure 3:**
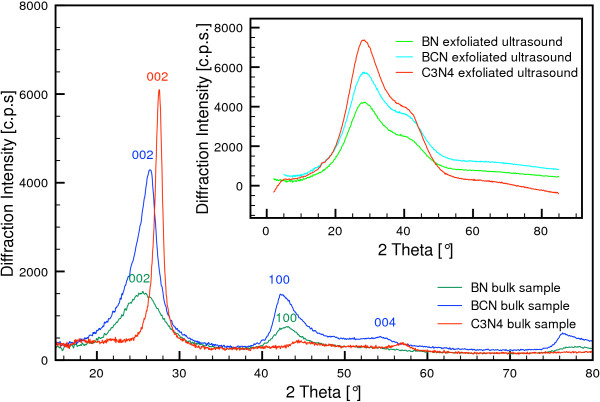
**XRD patterns of bulk synthetic samples of h-BN, h-BCN, and g-C**_**3**_**N**_**4**_**.** The inset shows the patterns of the ultrasound-exfoliated samples.

The raw samples of molybdenite and tungstenite correspond to the card numbers 96-900-9145 and 96-901-2192 of the crystallography open database (COD), as seen in Figure 2. The inset presents the XRD spectra of the exfoliated MoS_2_ and WS_2_, recorded in a water suspension between two Mylar foils to avoid drying and restacking. Figure 3 shows the XRD patterns of the synthesized bulk h-BN, h-BCN, and g-C_3_N_4_ used for exfoliation. The bulk h-BN showed a diffraction line with 2*Θ* at 25.5° (002) and one line with a lower intensity at 42.7° (100), which are indexed on JC PDF card number 85-1068. The h-BCN sample corresponds to the JC PDF card number 52-0233, and the diffraction pattern consisted of three weak peaks at 26.4° (002), 42.3° (100), and 54.8° (004). The g-C_3_N_4_ possesses two diffraction lines, and it is widely accepted that g-C_3_N_4_ is based on tri-*s*-triazine building blocks [40]. The strongest peak at 27.65° is a characteristic interlayer stacking peak of aromatic systems, indexed for graphitic materials as the (002) peak. The small angle peak at 13.01°, corresponding to an interplanar distance of 0.676 nm, is indexed as (100), which is associated with interlayer stacking [35].

When the exfoliated samples were dried, all of the characteristic peaks for raw IAGs - MoS_2_, WS_2_, h-BN, h-BCN, and g-C_3_N_4_ - reappear. The positions of the diffraction line (002) with 2*Θ* for MoS_2_ and WS_2_ at 14.3°, for h-BN and h-BCN at 26.0°, and for g-C_3_N_4_ at 28.0° were used to calculate the particle size and interlayer spacing (*d*_002_). To compare the crystal structures of the synthesized bulk and exfoliated samples, the crystallite sizes and interlayer spacing (*d*_002_) were calculated using the Scherrer equation in the High-Score Plus v. 3.0a program. The results are presented in Additional file 1: Table S1.

The dependence of the interlayer distance (*d*_002_) on the degree of unidimensional disorder, *γ*, in graphite-like BN was determined. It was established that in the perfectly ordered structure with *γ* = 0, *d*_002_ is equal to 0.333 nm. The value of *d*_002_ increased uniformly with an increase in *γ*; for *γ* = 1, the determined value of *d*_002_ is 0.343 nm [41]. The MoS_2_, WS_2_, and g-C_3_N_4_ interlayer spacing was 0.313 nm. The h-BCN interlayer spacing was determined to be approximately 0.335 nm [42] or approximately 0.35 nm [43], which is close to the typical *d*_002_ spacing in hexagonal structures and slightly longer than the distance in h-BN and graphite. In our case, the interlayer spacing was calculated to be 0.349 nm for bulk h-BN (1:3) and 0.341 nm for bulk h-BCN.

After exfoliation, wider interlayer spacings were expected, as was observed in the exfoliation of graphite [29]. However, as is evident from Additional file 1: Table S1, the value of *d*_002_, depending upon the number of layers, decreases to a value of approximately 0.31 nm. Banhart [44] observed a similar reduction in the spacing of graphene layers in carbon onions and interpreted the reduction as a compression and the transition of orbitals from sp^2^ to sp^3^. In the Fe_3_C encapsulated inside chain-like carbon nanocapsules, the smaller spacing of the graphene layers is related to the Fe_3_C particle. The bonding between the graphene layers and the Fe_3_C particle may contribute to the transition of orbitals from sp^2^ to sp^3^. The same effect - decreasing of *d*-spacing - was due to the interaction of the energetic particles with the carbon nanostructures [45].

In our case, the reduction of *d*-spacing is most likely due to the compression pressure caused by the collapse of the cavitation bubbles. Additional file 1: Figures S1 and S3 show high-resolution transmission electron microscopy (HRTEM) micrographs of exfoliated MoS_2_ and WS_2_ sheets that were obtained using ultrasound-assisted exfoliation. The *d*-spacing of MoS_2_ (0.639 nm) and WS_2_ (1.195 nm) corresponds with the (002) plane of the PDF 02-1133 card and the (205) plane of the PDF 08-0237 card, respectively.

Using the Miller-Bravais indices (*hkil*) for layered materials such as graphene, each set of diffraction spots exhibited an inner hexagon that corresponds with a (1-110) index and an outer hexagon that corresponds with a (1-210) index. The intensity profiles of the graphene diffraction patterns could therefore be used to determine the number of layers in the graphite sheet. The relative intensities of the diffraction spots in the inner and outer hexagons were shown to be equivalent to single-layer graphene, while the relative intensities of the spots in the outer hexagon were shown to be twice that of the spots in the inner hexagon for bilayer graphene [46]. The SAED of the prepared IAGs (MoS_2_ and WS_2_) are presented in Additional file 1: Figures S2 and S4, which shows the as-expected typical sixfold symmetry for the hexagonal forms of IAGs. The intensity of a line section through the (1-210), (0-110), (-1010), and (-2110) spots is shown in the inset figure. The inner (0-110)- and (-1010)-type reflections are more intense than the outer (1-210)- and (-2110)-type reflections, which is consistent with isolated single layers of IAGs.

HRTEM observations of the ultrasound-exfoliated h-BN and h-BCN (see Additional file 1: Figures S5 and S7) revealed a small fraction of an amorphous part in the material. By careful examination, we have found that besides the boron nitride nanocrystals with a size of approximately 2 to 3 nm, some amorphous domains were formed with an average diameter of 5 to 10 nm and are inter-layered within the crystalline domain. The *d*-spacings of the crystalline domains were found to be 0.345 and 0.366 nm for h-BN and h-BCN, respectively, which correspond to the (002) plane. Additional file 1: Figures S6 and S8 present SAED of synthesized h-BN and h-BCN, which confirms the results from XRD diffraction analysis.

TEM images and SAED of g-C_3_N_4_ are shown in Additional file 1: Figures S9 and S10. The sixfold symmetry is clearly visible, and Bravais-Miller (*hkil*) indices are used to label the diffraction peaks. Interestingly, the diffraction intensities of the inner spots (0-110) and (-1010) are always lower than those of the outer spots (1-210) and (-2110). This type of reflection would correspond to bilayer g-C_3_N_4_[47].

**Figure 4:**
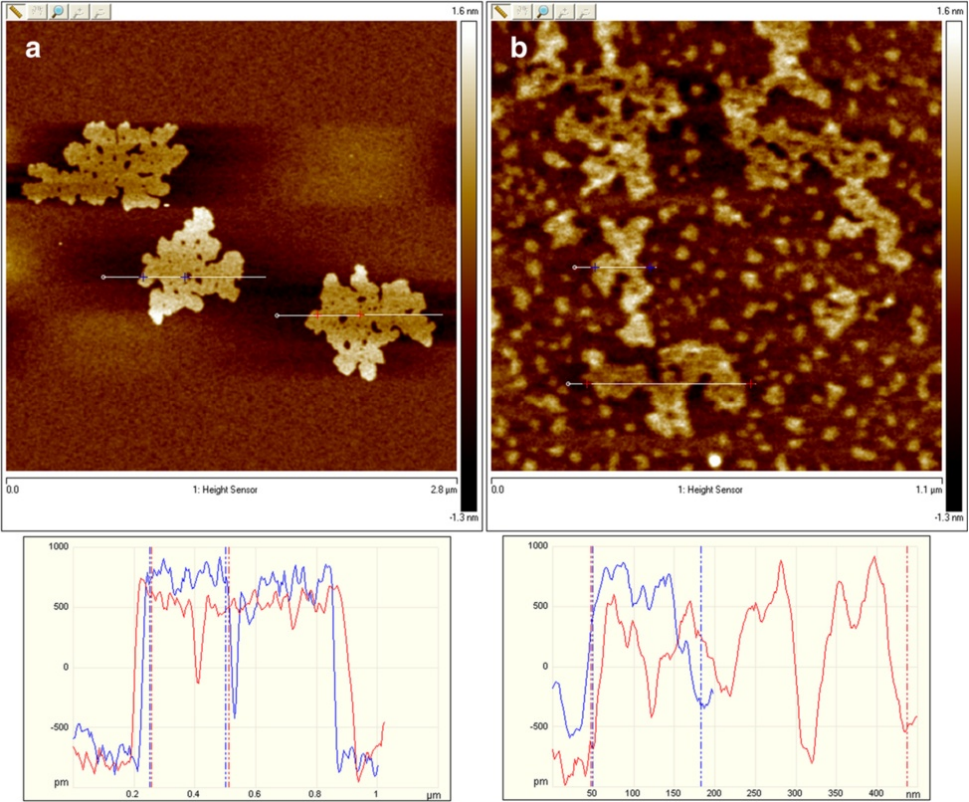
**AFM images and analysis of exfoliated MoS**_**2 **_**formed via (a) dimethylformamide and (b) an alkaline solution of potassium manganate.**

**Figure 5:**
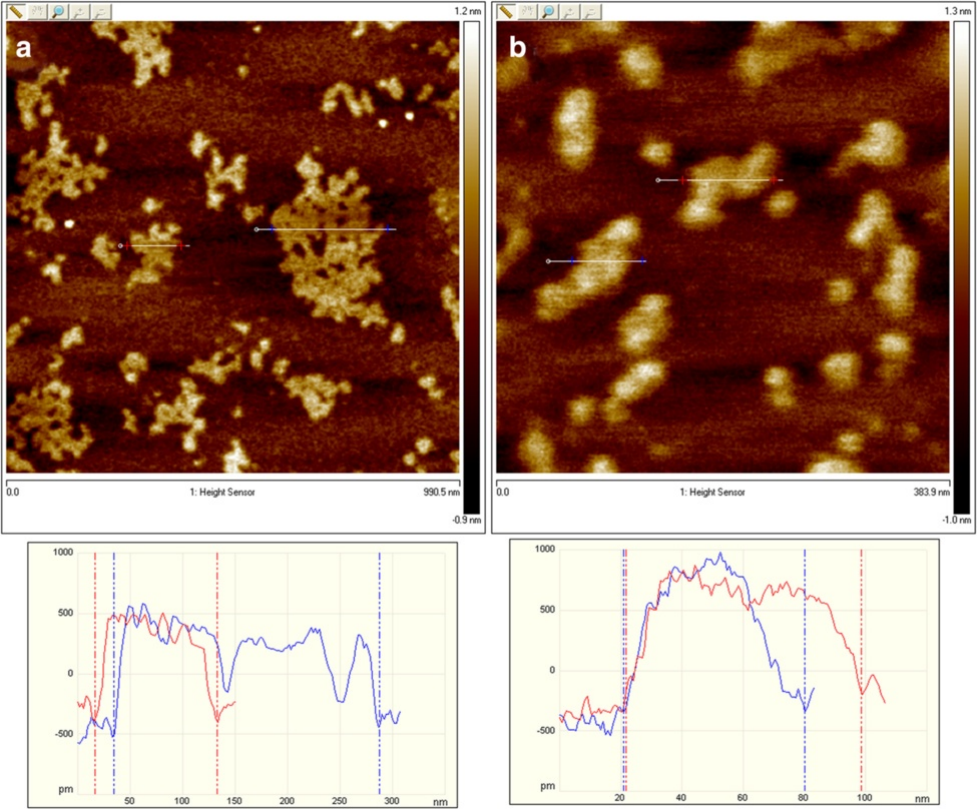
**AFM images and analysis of exfoliated WS**_**2 **_**in (a) dimethylformamide and (b) an alkaline solution of potassium manganate.**

**Figure 6:**
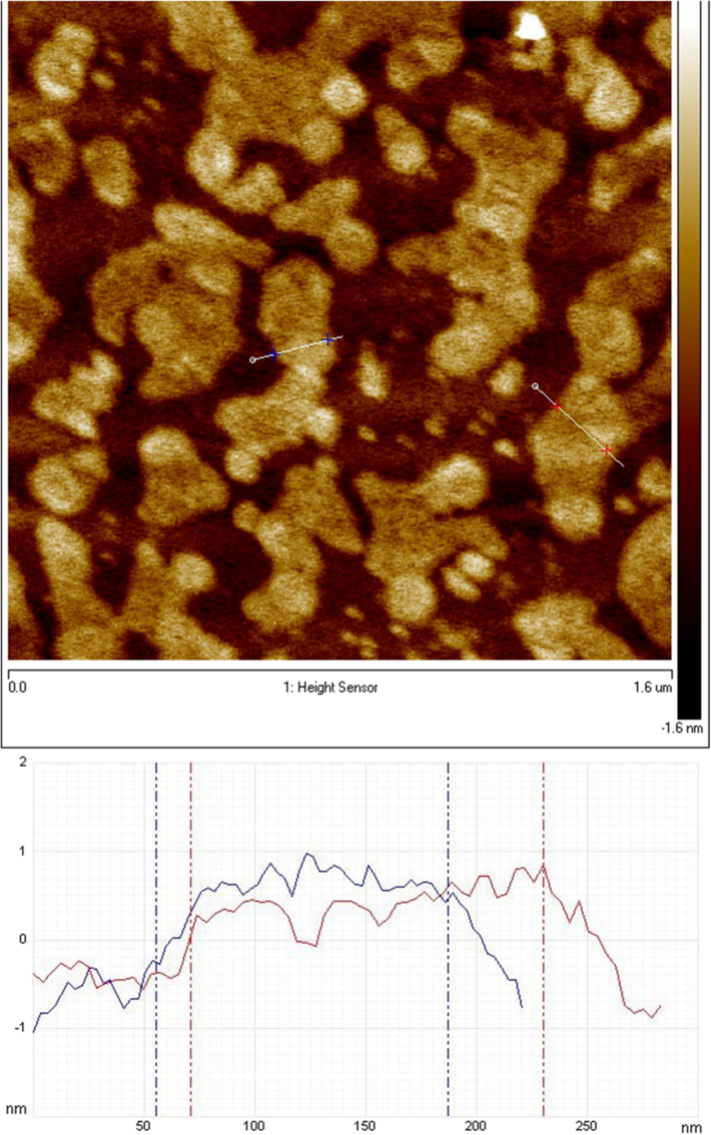
AFM image and analysis of exfoliated h-BN.

**Figure 7:**
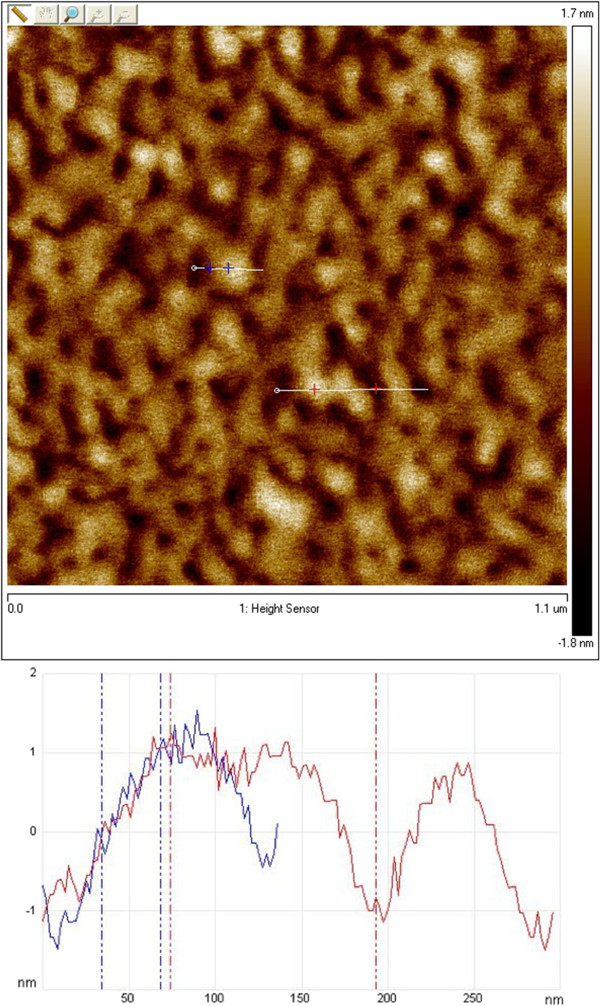
AFM image and analysis of exfoliated h-BCN.

**Figure 8:**
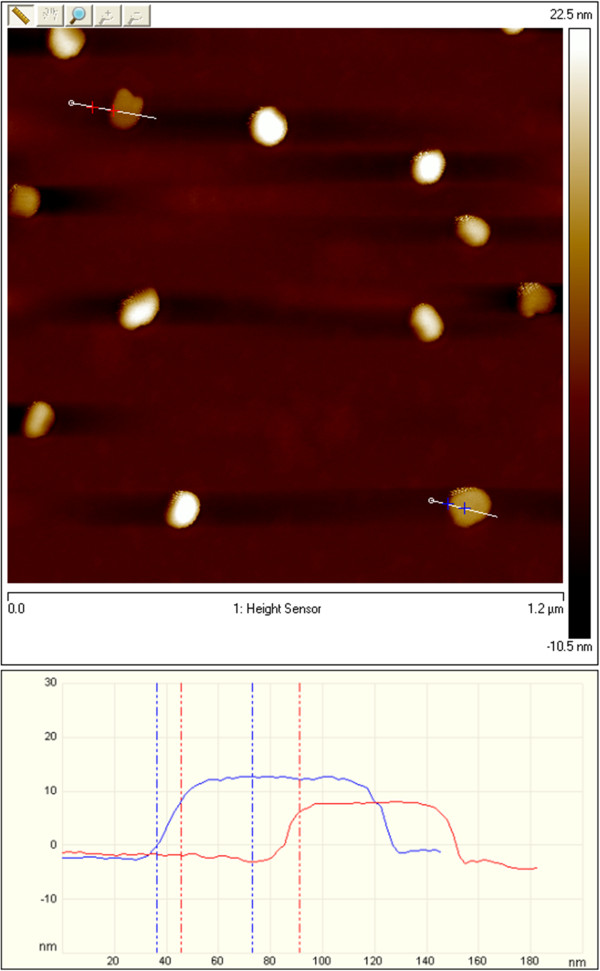
AFM image and analysis of chemically exfoliated h-BN.

The definite proof of the presence of exfoliated IAG sheets was provided by AFM, which can determine the height and therefore the number of layers. Figures 4 and 5 show the typical tapping-mode AFM images of MoS_2_ and WS_2_ exfoliated sheets using (a) dimethylformamide (DMF) and (b) the mixture of KMnO_4_ and KOH, which were deposited on a mica substrate. Cross-sectional analysis shows that the exfoliated MoS_2_ sheet had a thickness of approximately 0.7 nm and a lateral size of approximately 0.5 × 1.0 μm. Similarly, the exfoliated WS_2_ sheets possess a thickness of approximately 0.7 to 1 nm and a size of 80 to 100 nm. Thus, the conclusion from the observation of exfoliated WS_2_ and MoS_2_ is that single-layered sheets were achieved. This result is consistent with the aforementioned TEM observation. Figures 6 and 7 present AFM images of ultrasonically exfoliated h-BN and h-BCN. As seen in these figures, power ultrasound provided very uniformly delaminated materials. The analysis of the height profiles of both h-BN and h-BCN indicated that the thickness of the sheets is approximately 1 nm. This would note that the treated bulk-layered material provided mostly single (or double) sheets [48]. An important fact to emphasize is the height uniformity of the particles (clearly visible from the color scale) in the selected spots of the samples in the AFM analysis. Power ultrasound can therefore be suitable for larger scale production of homogeneous samples. The quality of the exfoliation by ultrasonic waves is evident in the comparison with chemically delaminated BN produced by the modified Hummers method [36]. As seen in the picture from the AFM microscope (see Figure 8), chemical delamination provided mostly 10-nm-thick particles of h-BN.

**Figure 9:**
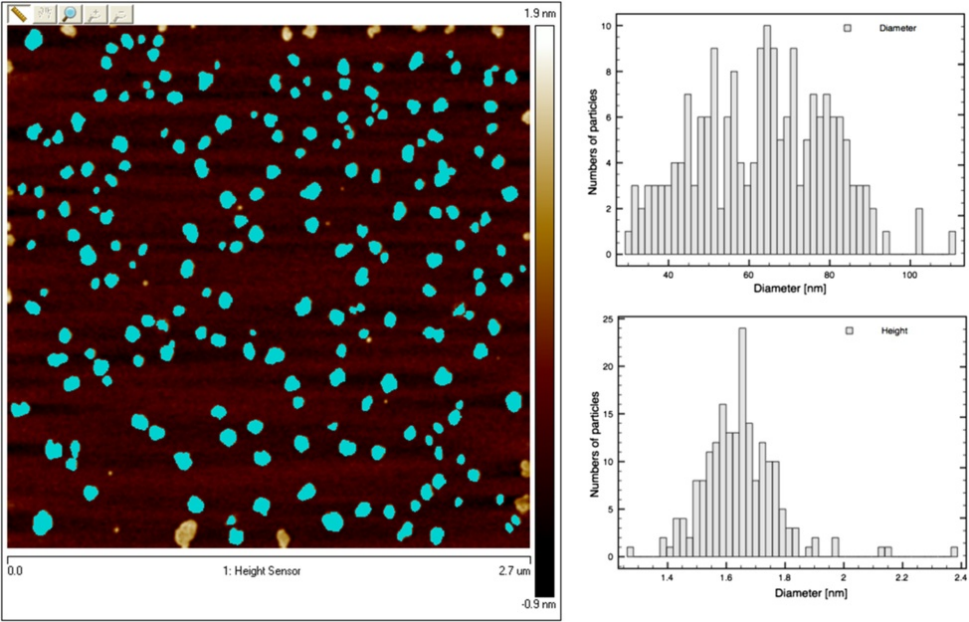
**AFM images and analysis of exfoliated g-C**_**3**_**N**_**4**_**.**

**Figure 10:**
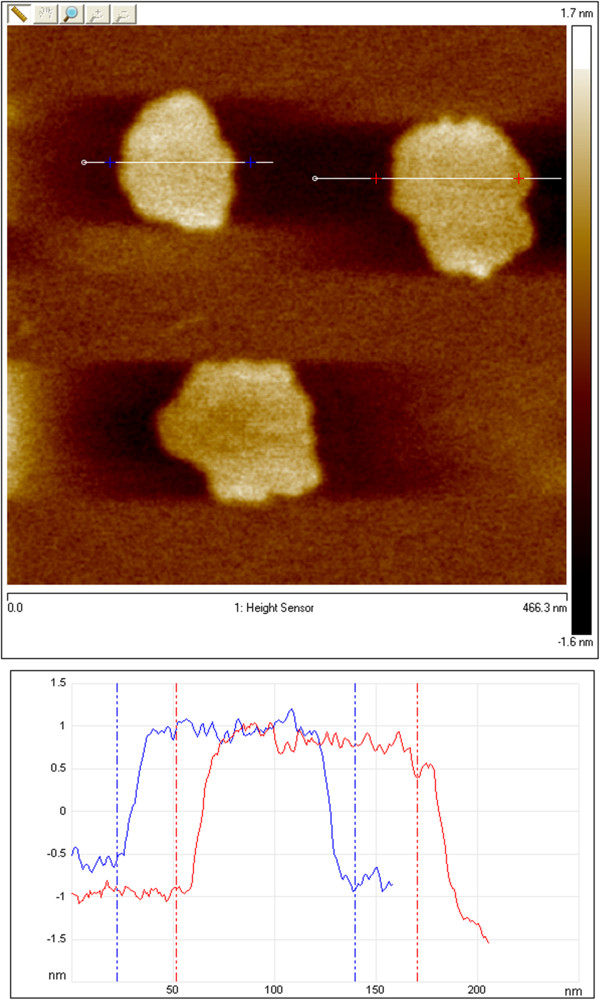
**High-resolution AFM image and analysis of exfoliated g-C**_**3**_**N**_**4**_**.**

The AFM image of exfoliated g-C_3_N_4_ in ethylene glycol is shown in Figure 9. From the image analysis, it is clear that the exfoliated sample formed particles of 60 to 80 nm in size with heights of approximately 1.6 nm. A high-resolution AFM image is presented in Figure 10. Cross-sectional analysis showed that the exfoliated g-C_3_N_4_ sheet has a thickness of approximately 0.1 nm and the sheet has a size of approximately 80 × 100 nm. These results correspond with the results from SAED for bilayer particles.

Zhi et al. [49] presented exfoliation of bulk h-BN in dimethylformamide by sonication for 10 h with subsequent centrifugation to remove residual large-sized BN particles. Approximately 0.5 to 1 mg of h-BN nanosheets could be routinely obtained from 1 g of the bulk h-BN powder; this corresponds to a yield of exfoliation of approximately 1%. The liquid exfoliation of layered materials [50,51] provided similar yields. All the aforementioned cited exfoliation methods improve yields by countless repetition of exfoliation with the necessary intermediate operations (centrifugation) that isolate exfoliated products from the initial suspension. The resulting product is a diluted dispersion of the nanosheets in a suitable solvent.

Here, the reported method using the high-power ultrasound produced a concentrated colloidal dispersion of nanosheets by one-step sonication; the product possesses a relatively homogeneous distribution of the few- or monolayers, as seen in the AFM images. By this method, large quantities of the colloidal dispersion of nanosheets are readily available as a precursor (for example, for the preparation of composites) and can be produced in a short time. Using an alkaline medium to prepare exfoliated IAGs could be an important shift in the preparation of these materials. Using alkaline solutions for ultrasonic preparation could exclude hydrophobic organic solvents and consequently contamination by organic residuals and undesired functionalization of the nanosheets. Furthermore, the product prepared in an alkaline environment could possess a different reactivity, thanks to possibly different composition of surface functional groups. However, further investigations of this proposed method are necessary.

## Conclusions

The method of exfoliation in a pressurized batch ultrasonic reactor allows for the preparation of few- and monolayered colloidal dispersions of IAG particles without intercalation. The quality and quantity of the exfoliation depends upon appropriate selection of the reaction conditions (intensity of ultrasound, the reaction time, the pressure in the reactor, etc.). Strong aprotic solvents (NMP, DMF, DMSO, etc.) are used for the preparation of monolayered IAGs in a hydrophobic environment. The method of exfoliation of IAGs that is based on the intercalation of potassium manganate in an alkaline environment in the presence of high-intensity ultrasound is suitable for hydrophilic applications with a good dispersibility of the IAGs in water. This non-oxidative method allows for the preparation of exfoliated IAGs of high purity with a minimum content of undesirable functional groups.

## Competing interests

The authors declare that they have no competing interests.

## Authors’ contributions

VS was the main author of the work, performed the syntheses, and coordinated all characterization. MS was responsible for the electron microscopy, JH for the AFM microscopy, and PE for the XRD measurement. All authors read and approved the final manuscript.

## Supplementary Material

Additional file 1 **Supplement information Table S1.** Integral breath, d-spacing and crystallite size of prepared samples IAGs. **Figure S1.** HRTEM of exfoliated MoS2. **Figure S2.** SAED of exfoliated MoS2. **Figure S3.** HRTEM of exfoliated WS2. **Figure S4.** SAED of exfoliated WS2. **Figure S5.** HRTEM of exfoliated h-BN. **Figure S6.** SAED of exfoliated h-BN. **Figure S7.** HRTEM of exfoliated h-BCN. **Figure S8.** SAED of exfoliated h-BCN. **Figure S9.** TEM of exfoliated g-C_3_N_4_. **Figure S10.** SAED of exfoliated g-C_3_N_4_. Click here for file
