# Supplementary material for: Ultrasound exfoliation of inorganic analogues of graphene
Source: Nanoscale Res Lett. 2014 Apr 5;9(1):167. doi: 10.1186/1556-276X-9-167 (PMC4022084; doi:10.1186/1556-276X-9-167)
Supplement: Additional file 1 — Supplement information Table S1. Integral breath, d-spacing and crystallite size of prepared samples IAGs. Figure S1. HRTEM of exfoliated MoS2. Figure S2. SAED of exfoliated MoS2. Figure S3. HRTEM of exfoliated WS2. Figure S4. SAED of exfoliated WS2. Figure S5. HRTEM of exfoliated h-BN. Figure S6. SAED of exfoliated h-BN. Figure S7. HRTEM of exfoliated h-BCN. Figure S8. SAED of exfoliated h-BCN. Figure S9. TEM of exfoliated g-C3N4. Figure S10. SAED of exfoliated g-C3N4. [file 1556-276X-9-167-S1.pdf]

Supplement Information

Ultrasound Exfoliation of Inorganic Analogues of Graphene

Václav Štengl*, Jiří Henych, Michaela Slušná

Department of Solid State Chemistry, Institute of Inorganic Chemistry AS CR v.v.i.

250 68 Řež, Czech Republic

e-mail:stengl@iic.cas.cz

Table S1. Integral breath, d-spacing and crystallite size of prepared samples IAGs

Sample	Peak Position 2Θ [°]	Integral breadth 2Θ[°]	d-spacing [nm]	Cryst. size [nm]
MoS2	14.37	0.19	0.665	826.1
MoS2_exf	28.34	15.11	0.313	5.47
WS2	14.34	0.42	0.618	259.0
WS2_exf	28.47	15.06	0.313	5.99
h-BN	39.54	6.95	0.349	13.20
h-BN_exf	28.03	14.22	0.317	6.37
h-BCN	26.11	4.40	0.341	21.00
h-BCN_exf	28.25	12.48	0.315	7.33
g-C3N4	27.48	1.63	0.324	58.18
g-C3N4_exf	28.53	15.17	0.313	5.96

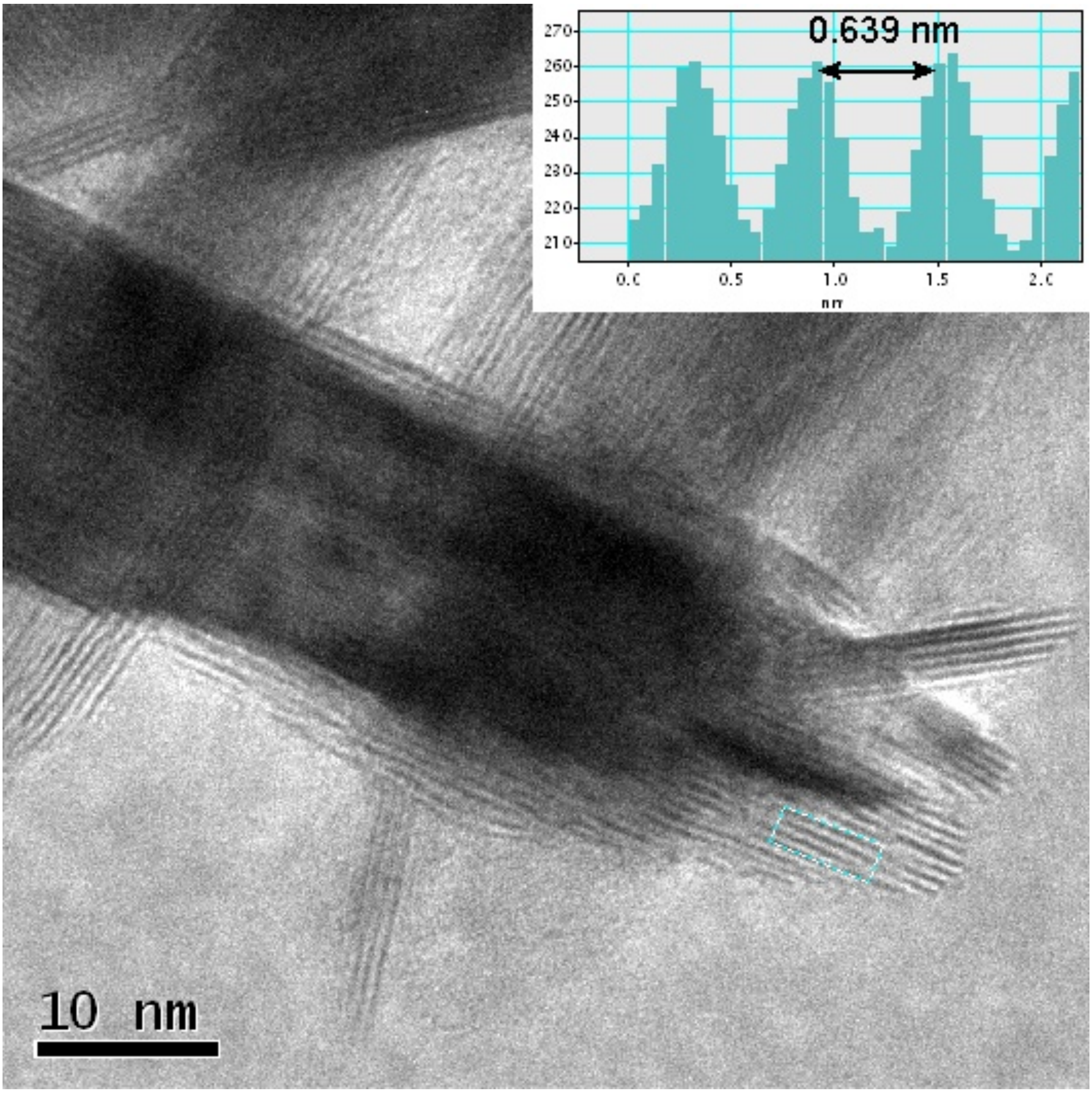

Figure S1. HRTEM of exfoliated MoS2

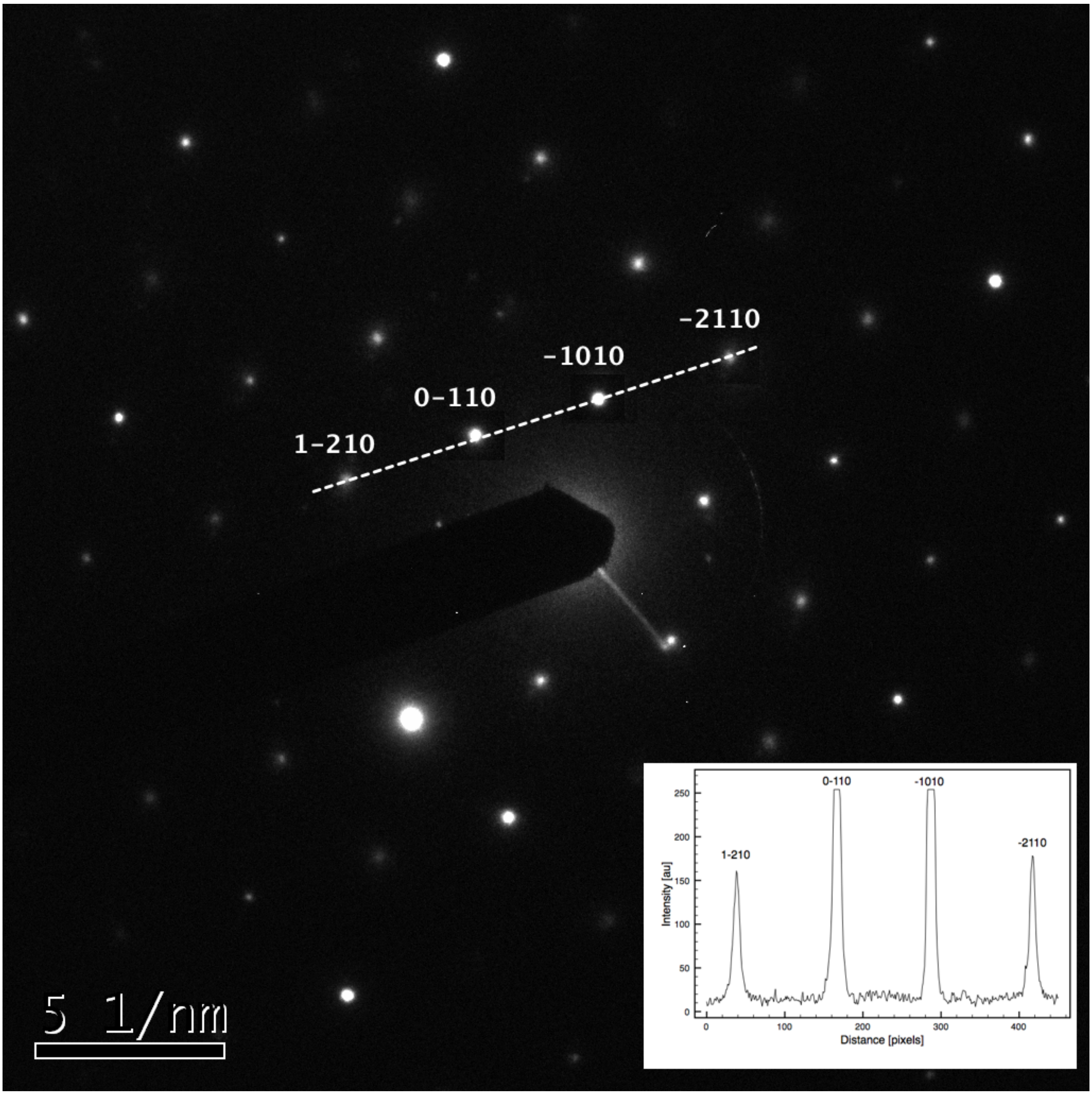

Figure S2. SAED of exfoliated MoS2

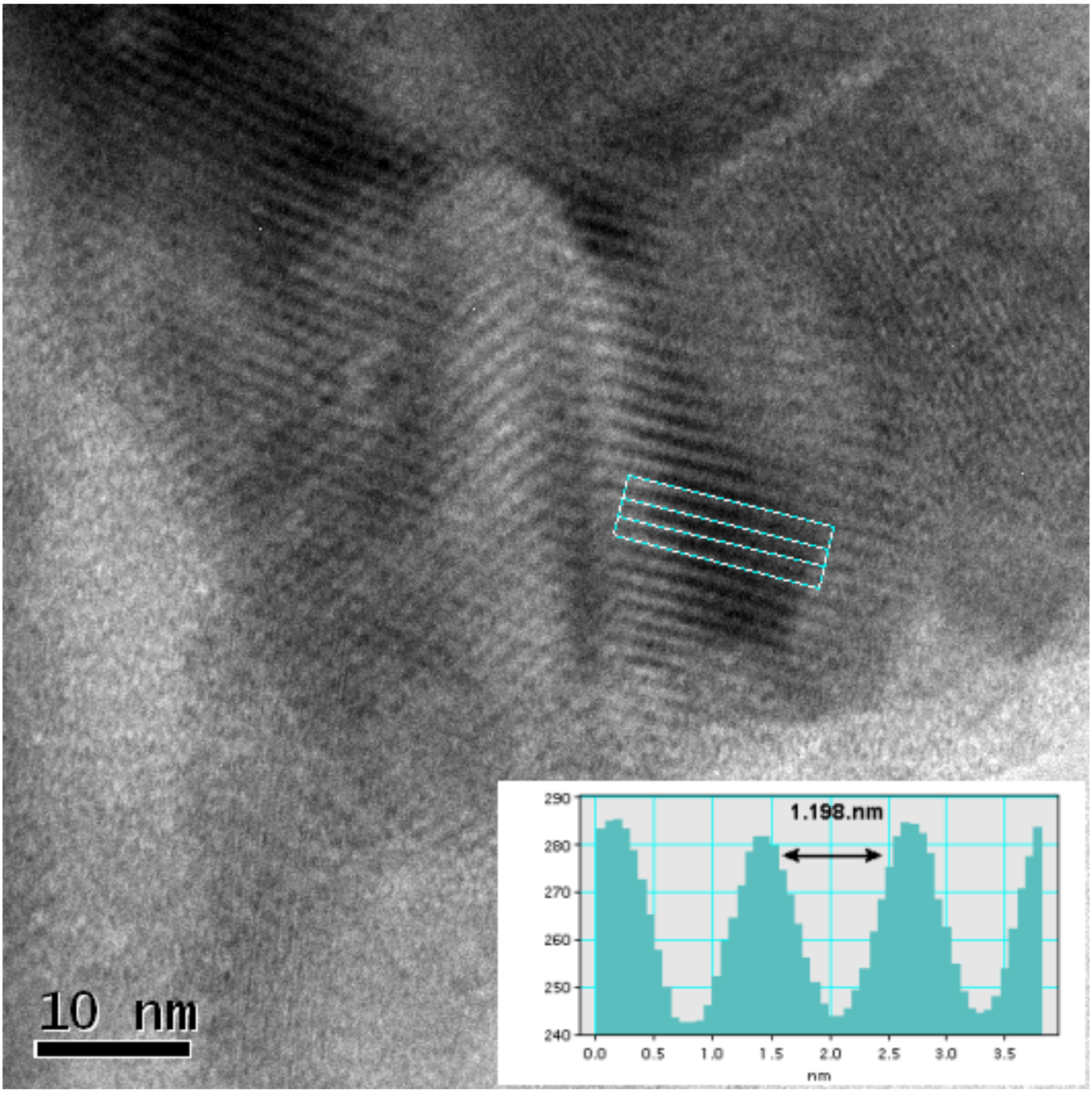

Figure S3. HRTEM of exfoliated WS2

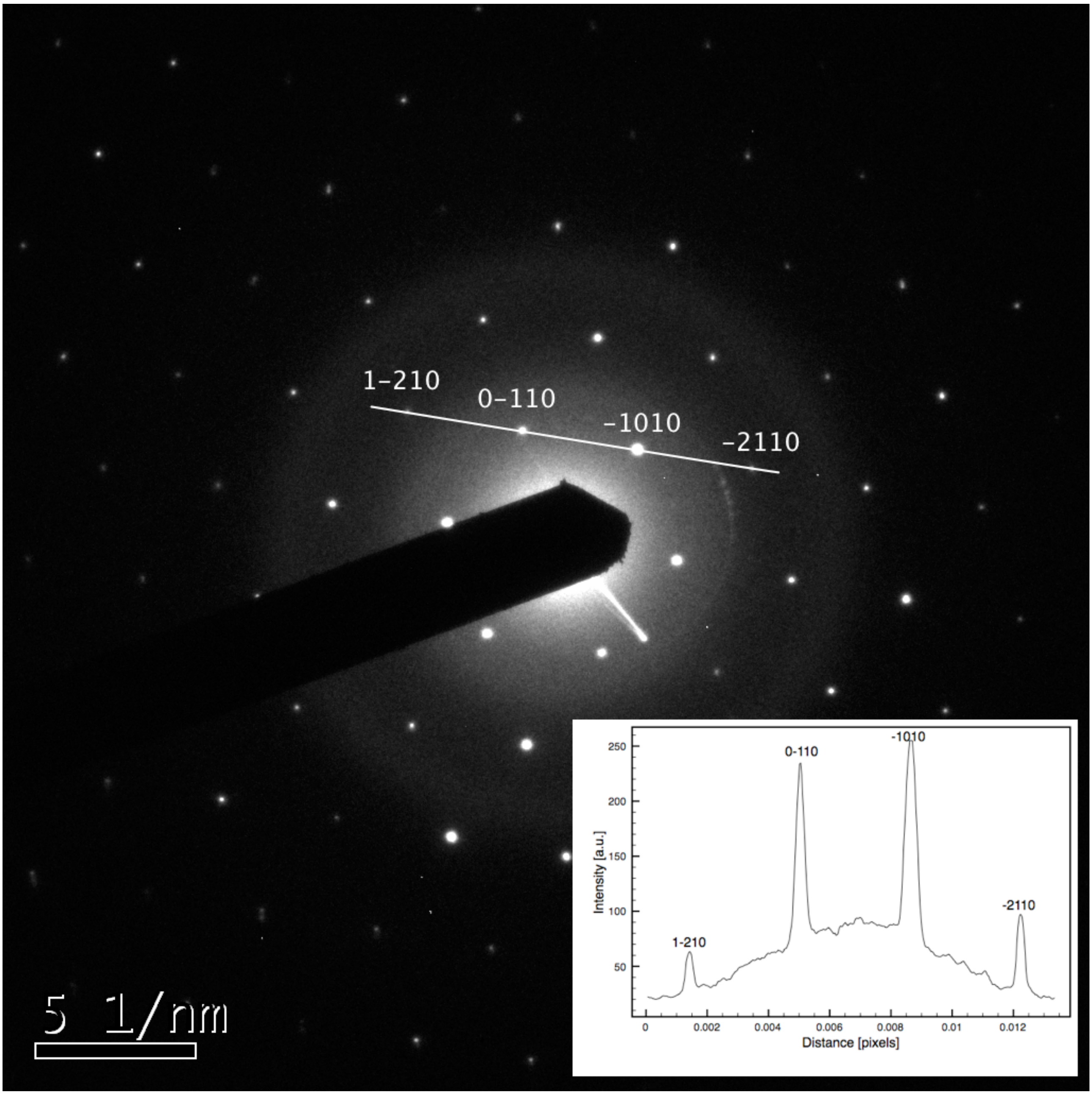

Figure S4. SAED of exfoliated WS2

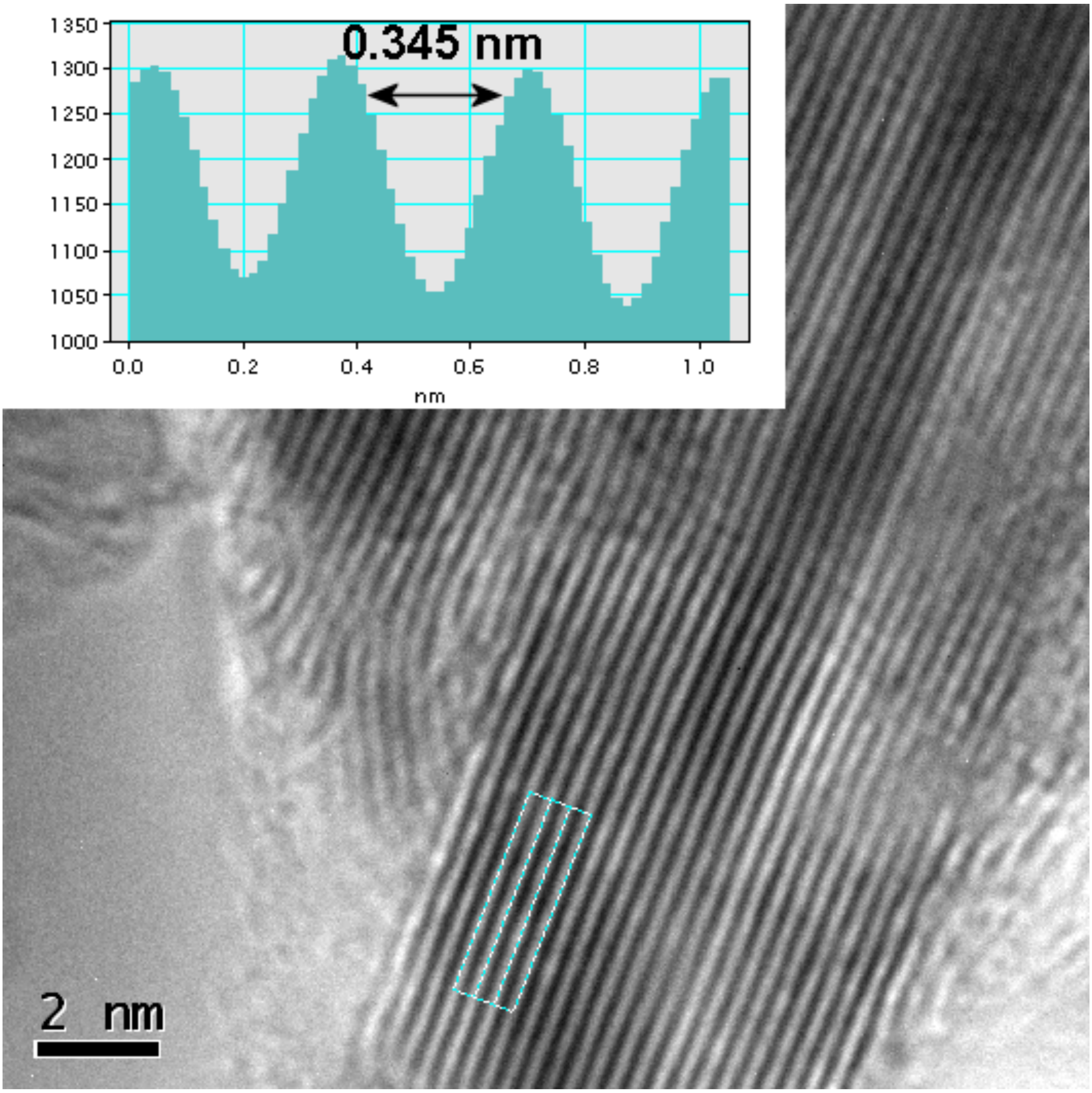

Figure S5. HRTEM of exfoliated h-BN

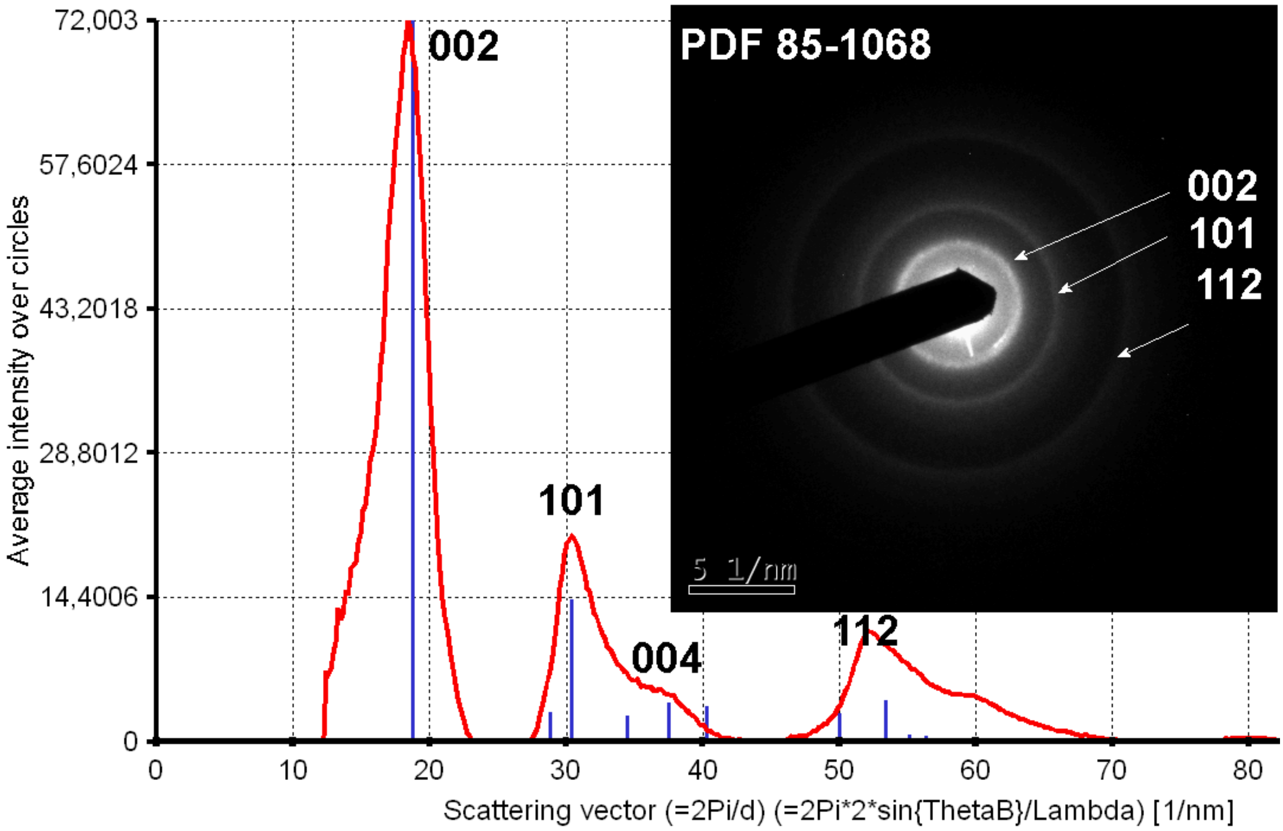

Figure S6. SAED of exfoliated h-BN

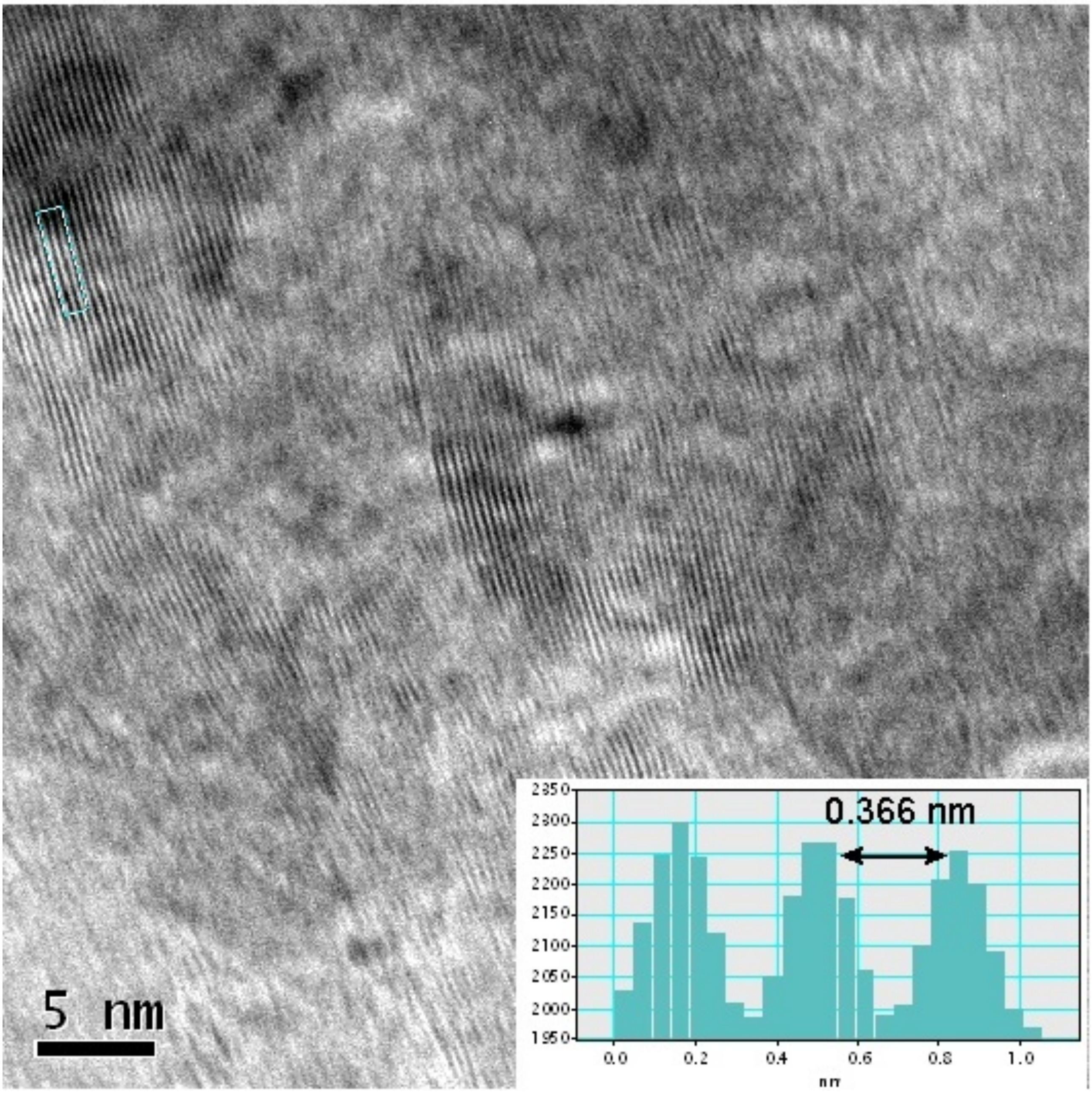

Figure S7. HRTEM of exfoliated h-BCN

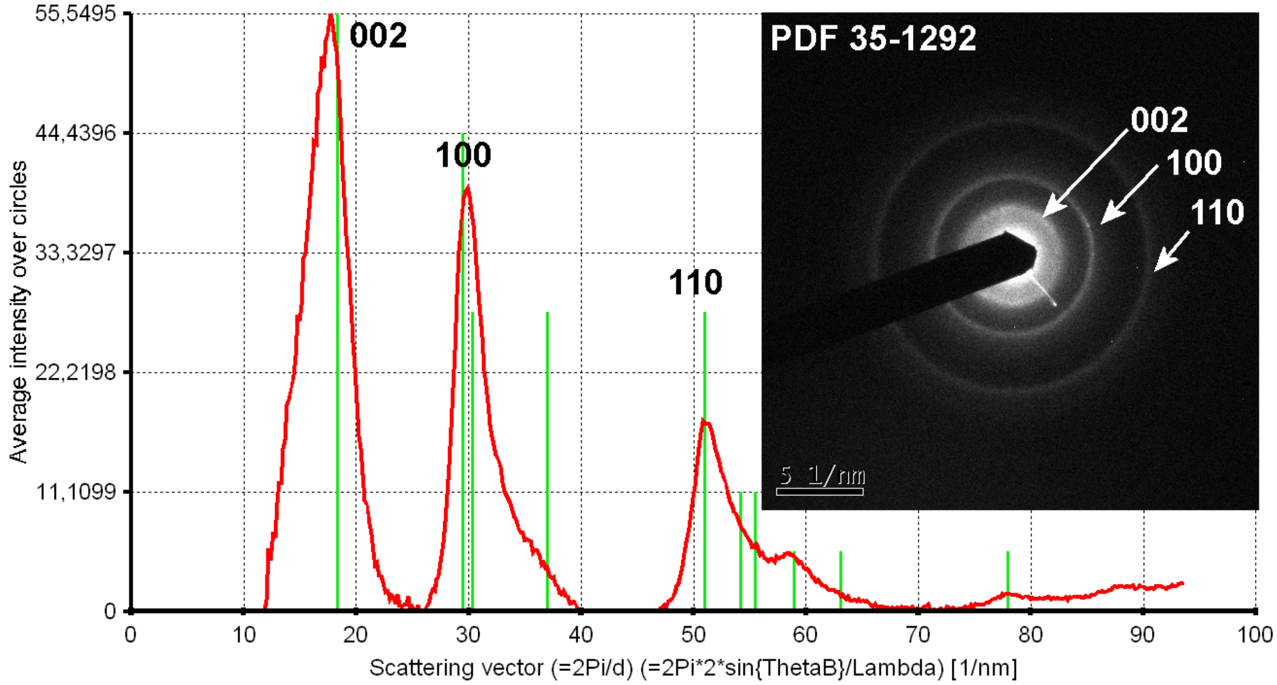

Figure S8. SAED of exfoliated h-BCN

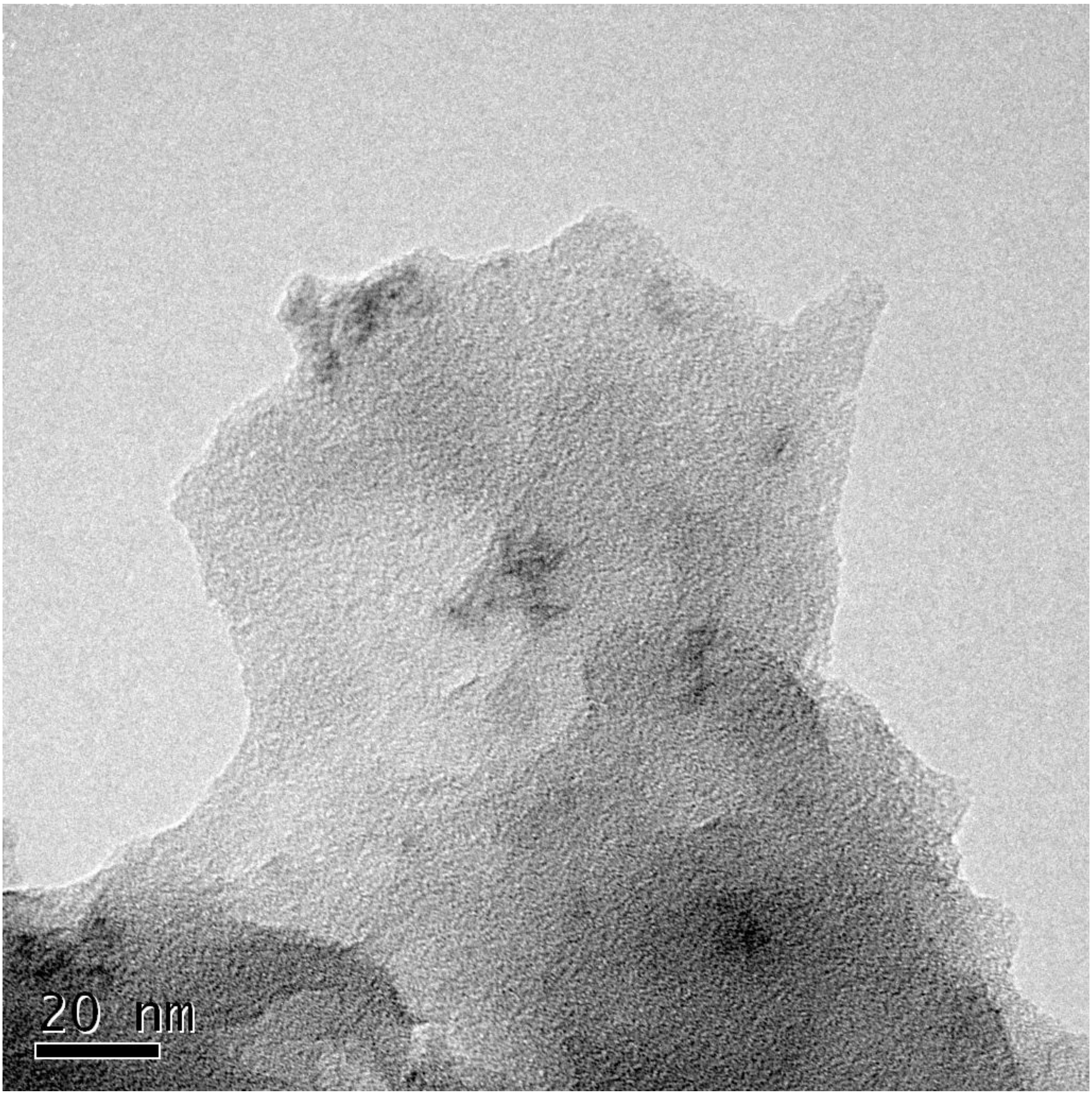

Figure S9. TEM of exfoliated g-C_3N_4

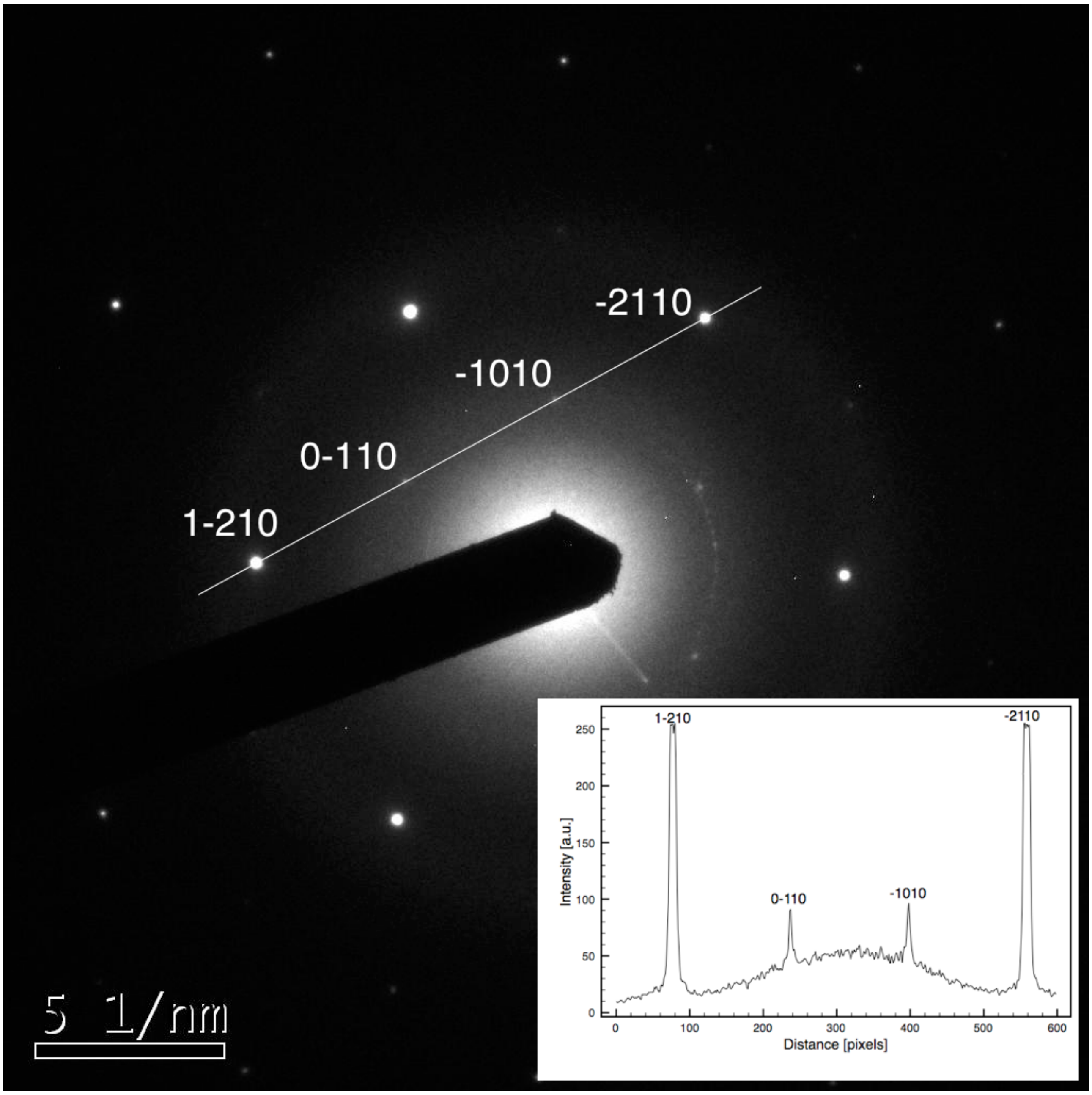

Figure S10. SAED of exfoliated g-C_3N_4
